# Research on the Ignition Height and Reaction Flame Temperature of PTFE/Al/Si/CuO with Different Mass Ratios of PTFE/Si

**DOI:** 10.3390/ma14133464

**Published:** 2021-06-22

**Authors:** Xuan Zou, Jingyuan Zhou, Xianwen Ran, Yiting Wu, Ping Liu, Wenhui Tang, Pengwan Chen, Haifu Wang

**Affiliations:** 1 College of Liberal Arts and Sciences, National University of Defense Technology, Changsha 410073, China; zouxuan14@nudt.edu.cn (X.Z.); zhoujingyuan19@nudt.edu.cn (J.Z.); liuping14@nudt.edu.cn (P.L.); 2 College of Aerospace Science and Engineering, National University of Defense Technology, Changsha 410073, China; wuyiting@nudt.edu.cn; 3 School of Mechatronical Engineering, Beijing Institute of Technology, Beijing 100081, China; pwchen@bit.edu.cn (P.C.); wanghf@bit.edu.cn (H.W.)

**Keywords:** PTFE/Al/Si/CuO, flame duration, ignition height, flame spectrum, flame temperature

## Abstract

Recent studies have shown that the energy release capacity of Polytetrafluoroethylene (PTFE)/Al with Si, and CuO, respectively, is higher than that of PTFE/Al. PTFE/Al/Si/CuO reactive materials with four proportions of PTFE/Si were designed by the molding–sintering process to study the influence of different PTFE/Si mass ratios on energy release. A drop hammer was selected for igniting the specimens, and the high-speed camera and spectrometer systems were used to record the energy release process and the flame spectrum, respectively. The ignition height of the reactive material was obtained by fitting the relationship between the flame duration and the drop height. It was found that the ignition height of PTFE/Al/Si/CuO containing 20% PTFE/Si is 48.27 cm, which is the lowest compared to the ignition height of other Si/PTFE ratios of PTFE/Al/Si/CuO; the flame temperature was calculated from the flame spectrum. It was found that flame temperature changes little for the same reactive material at different drop heights. Compared with the flame temperature of PTFE/Al/Si/CuO with four mass ratios, it was found that the flame temperature of PTFE/Al/Si/CuO with 20% PTFE/Si is the highest, which is 2589 K. The results show that PTFE/Al/Si/CuO containing 20% PTFE/Si is easier to be ignited and has a stronger temperature destruction effect.

## 1. Introduction

In recent studies, energy release characteristics of Polytetrafluoroethylene (PTFE)/Al reactive material have been widely recognized [1,2,3]. Compared with TNT, the calorific value of the same volume of PTFE/Al (mass ratio 73.5/26.5) is about 2.4 times that of TNT, which indicates that the PTFE/Al reactive material may become a new type of high-energy explosive [4].

Yang [5] studied the influence of the molding–sintering process on the mechanical properties of PTFE/Al (mass ratio 73.5/26.5). It was found that the tensile strength of PTFE/Al ranged from 17 to 23 MPa, the elongation at break was 300% to 380%, the compressive strength could be as high as 160 MPa, and the typical density was 2.284 g/cm^3^. The high strength of PTFE/Al indicates that PTFE/Al can be developed into an energetic structural material. Huang [6] studied the damaging effect of PTFE/Al fragments through experiments and found that the damaging effect of reactive fragments was significantly better than inert fragments. It can be seen from the above that PTFE/Al has higher structural strength and calorific value compared with traditional explosives. Simultaneously, PTFE and Al have a wide range of sources and low manufacturing costs. Therefore, PTFE/Al can be widely used in the military as high-energy explosives, reactive fragments, and reactive charge jets [7,8,9].

PTFE/Al was insensitive to traditional initiation methods and needed to be loaded at a high strain rate (10^3^ s^−1^) or high-speed impact (generally greater than 300 m/s) to initiate a chemical reaction and release a large amount of energy [10]. Some scholars added different materials to PTFE/Al to increase the reactive material density and sensitivity in order to promote energy release. Cai [11,12,13] prepared a higher density reactive material (PTFE/Al/W) and studied the mechanical properties of the reactive material containing tungsten particles of different sizes (1 μm and 44 μm). Ding [14] studied PTFE/Al/CuO reactive material and showed that the density of CuO was higher than PTFE/Al, and the combustion reaction rate of the Al/CuO was higher than that of Al/PTFE. Therefore, the addition of CuO powder adjusted the reactive material’s density and promoted the chemical reaction between the components of the reactive material. Zhou [15] found that adding Al/CuO thermite materials or using nano-scale PTFE and Al materials can significantly improve the reactive material sensitivity in drop hammer tests. Ran [16] studied PTFE/Al with B, Si, and CuO, respectively, and found that PTFE/Al/Si can produce higher pressure, indicating that it has a more vital destructive ability.

Based on the literature mentioned above, we know that there has been much work on preparation and energy release testing of reactive materials. These works have shown significant results, which provided a basis for the research of this article. In this study, the ignition height and reaction flame temperature of PTFE/Al/Si/CuO were obtained by the drop hammer and spectrophotometer system to study the influence of different mass ratios of PTFE/Si on the energy release characteristics of PTFE/Al/Si/CuO.

## 2. Experimental Setup

### 2.1. Formulation Design of Reactive Materials

In this study, the reactive materials were based on PTFE/Al. CuO powder was added to increase the density and energy release capacity. Different ratios of PTFE/Si were added to study the effect of PTFE/Si on PTFE/Al/Si/CuO. The relative molecular mass, density, powder diameter, and manufacture of raw materials in this study are shown in Table 1.

The main chemical reactions during the energy release process of the specimens include:

4Al + 3C_2_F_4_ → 4AlF_3_ + 6C (1)

2Al + 3CuO → Al_2_O_3_ + 3Cu (2)

Si + C_2_F_4_ → SiF_4_ + 2C (3)

It can be seen from the above equation that the energy release process of the specimens includes three kinds of reaction processes. Assuming that the material reacts completely, the mass ratios of PTFE/Al, Al/CuO, and PTFE/Si are determined according to the three equations’ coefficients. Moreover, 10% mass fraction of Al/CuO and four mass ratios of PTFE/Si were added to PTFE/Al, respectively, and the composition ratios of four specimens are shown in Table 2.

### 2.2. Specimen Preparation

The reactive materials were composed of PTFE, Al, Si, and CuO, but the affinity between PTFE and Al is poor. Rose [17,18] proposed a feasible method to enhance PTFE-based reactive materials, using coupling agents to improve the polymer and the filler’s interfacial bonding force. In this study, a coupling agent was selected for surface treatment of the metal powder to promote the bonding strength between the PTFE and Al surface. First, an amount of coupling agent (1% of the mass of Al powder) was dissolved into absolute ethanol. Then, the Al powder was added to the solution. After the solution stood for 1 h, the solution was heated and stirred until the solution completely evaporated. Finally, the material was placed in a vacuum drying oven and dried for about 6 h.

Different materials were poured into the QM-3SP04 planetary ball mill (manufactured by Nanjing NanDa Instrument Plant, Nanjing, China) after the drying was completed according to different mass ratios. The materials were ground and mixed for 1 h at a speed of 40 cycles per second. The uniformly mixed materials were taken out from the ball mill and sealed in plastic bags. The bags were placed in a constant temperature drying box to prevent oxidation and moisture.

According to the size of the specimen, we weighed 0.21 g of the mixed powder and poured it into a mold with a Φ6 mm hollow cylinder. The powder was pressed at a compressive pressure of 500 MPa for about 1–2 min. Then, the specimens were removed from the mold and placed for about 24 h in an ambient temperature, ambient pressure, and dry environment to release the residual stress.

**Figure 1:**
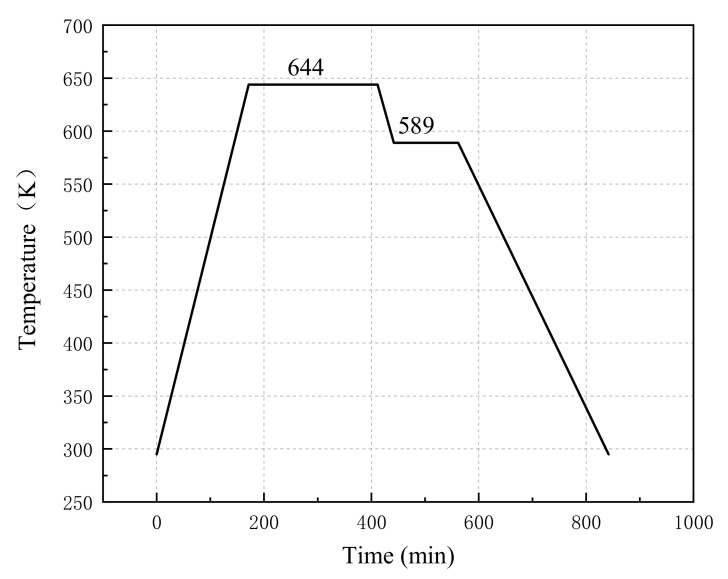
Sintering temperature curve of specimens.

**Figure 2:**
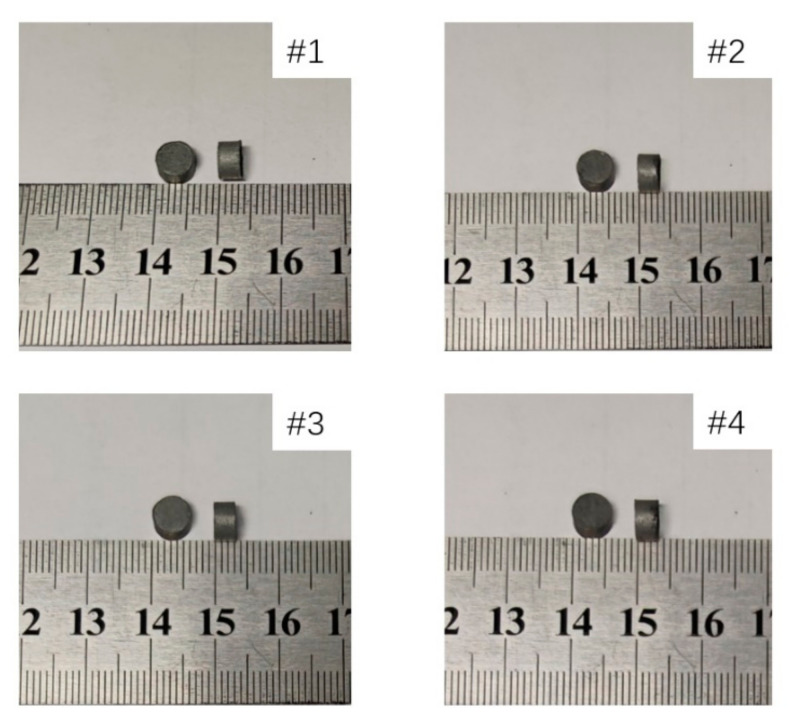
Shape and size of four different materials after sintering.

The specimens were put in the sintering furnace and sintered based on the patent of Nielson [19]. The reactive materials with different proportions were sintered separately. The temperature history of the sintering cycle is depicted in Figure 1. After sintering, the mass and size of the four specimens were measured and recorded in Table 3. The shape and size of the samples are shown in Figure 2.

### 2.3. Experimental Procedures

A drop hammer experiment was performed to ignite PTFE/Al/Si/CuO reactive materials to study the ignition height at the ambient temperature of 22 °C. The instrument had a drop mass of 10 kg, which could be released from a maximum height of 240 cm. The specimens were placed on the tester’s anvil without constraint and impacted directly by the drop hammer falling free. The experiment was carried out according to the method of gradually decreasing the drop height from 240 cm. A high-speed camera recorded the experimental process at a speed of 10,000 frames per second.

**Figure 3:**
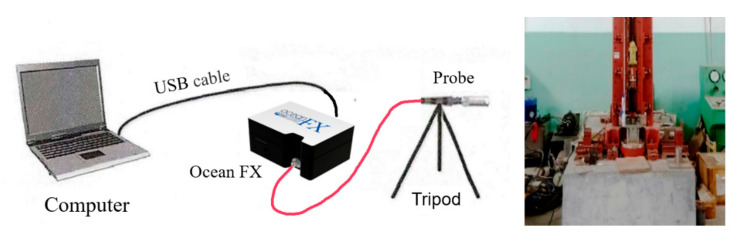
Schematic diagram of flame spectrum measuring system.

Ocean FX network high-speed spectrometer was used to record the spectrum of the reaction flame of the PTFE/Al/Si/CuO reactive material. An optical probe was aligned with the specimen, the other end of the optical was connected to the spectrometer, and the spectrometer was connected to a computer through a cable. Before the experiment, A halogen lamp was used to calibrate the spectrometer. In the experiment, the spectrometer was triggered to collect signals when releasing the drop hammer. The schematic diagram of the spectrum measurement system is shown in Figure 3. Information about the experimental instrument is shown in Table 4.

## 3. Results and Discussion

### 3.1. Calculation of Reactive Material Ignition Height

**Figure 4:**
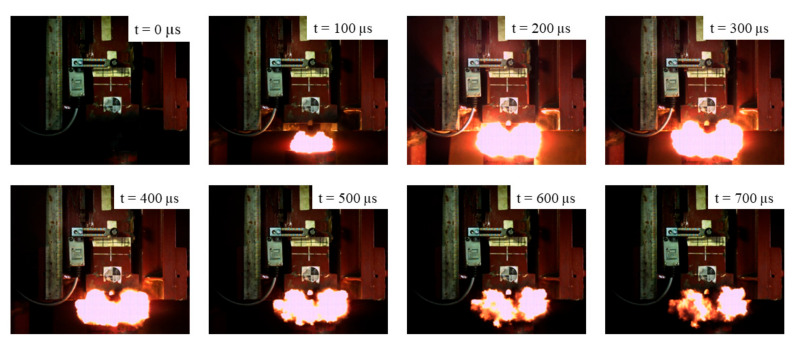
The images of the energy release process of the #1 material at a drop height of 240 cm.

The intensity of the reactive material reaction is proportional to the energy input by the drop hammer. Within a specific range, the higher the drop weight, the longer the reaction time. The ignition heights of the four kinds of specimens were calculated by the relationship between flame duration and drop height. The reaction process of the #1 material is shown in Figure 4 at a drop height of 240 cm.

Three experiments were performed under the same drop height to calculate the average flame duration. Table 5 records the average and standard deviation of the flame duration of the four specimens at different drop heights.

The results show that the flame duration of the four specimens ignited under the same drop is as follows: #2 > #3 > #1 > #4, which indicates that #2 is the easiest to ignite in the four specimens. The flame duration of the same sample increased with the increase of the drop height, and the flame duration increased rapidly at first, and then gradually slowed down.

According to the changing trend of flame duration with drop height, we chose the Equation (4) to fit the relationship between the flame duration and the drop height.

(4) y=Aln(Bx+C)+D

**Figure 5:**
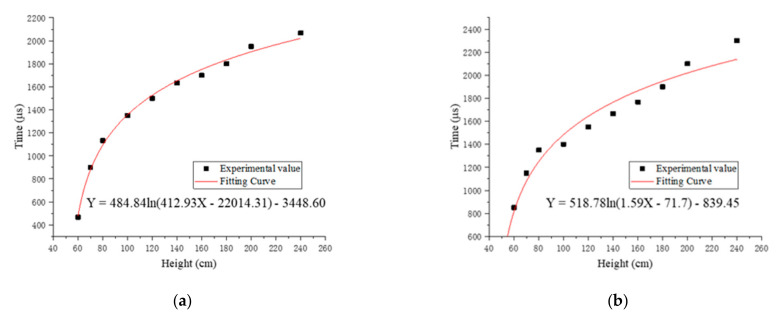

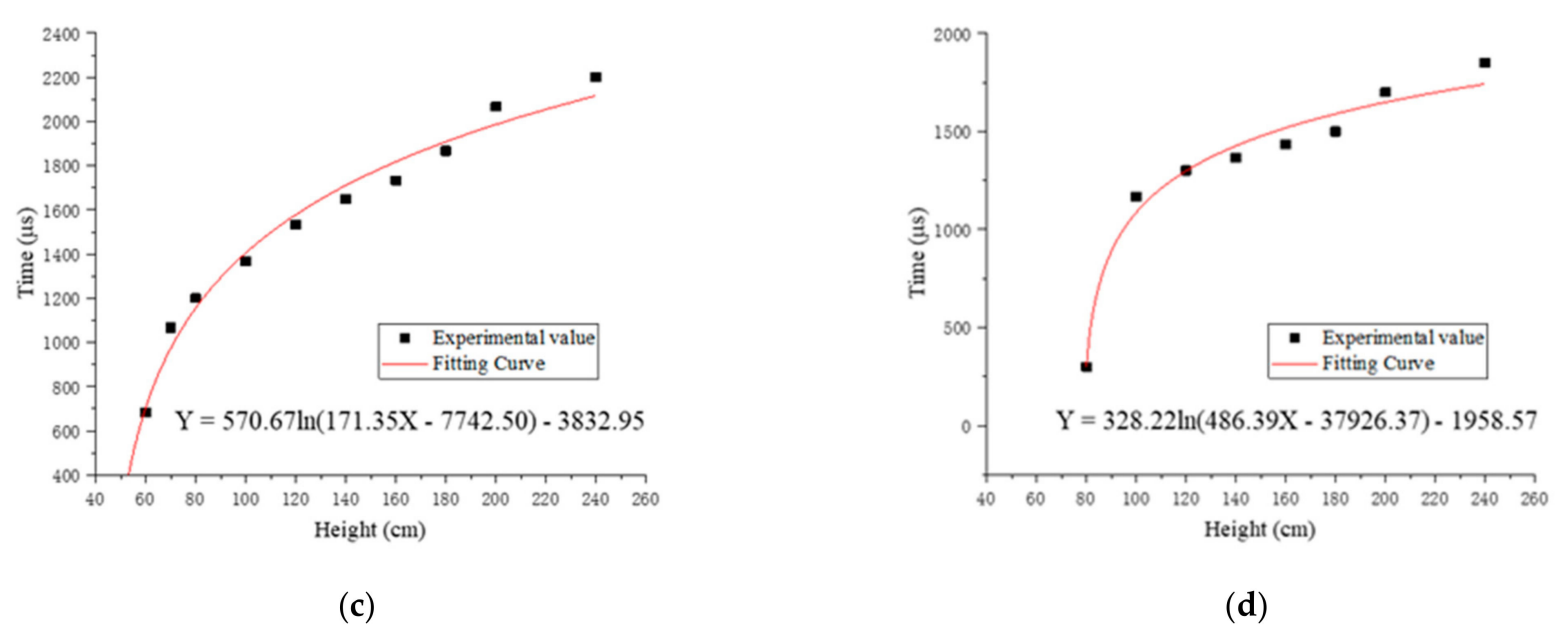
The fitting curves and functions of the relationship between the drop height and flame duration of four specimens: (**a**) fitting curve and function of #1; (**b**) fitting curve and function of #2; (**c**) fitting curve and function of #3; (**d**) fitting curve and function of #4.

In the equation, *A*, *B*, *C*, *D* are constants, *x* is the drop height, and *y* is the flame duration. The Levenberg–Marquardt optimization algorithm was used to fit the experimental value. Figure 5 shows the fitting curves and functions of the relationship between the dropping height and flame duration of four specimens.

The fitting flame duration of the four reactive materials at different drop heights calculated by the fitting function subtracted the experimental value, and then the errors were recorded, as shown in Table 6.

Equation (5) was used to calculate the square root of the sum of squared errors of the four reactive materials.

(5) $$\sigma =\sqrt{\frac{1}{N}\sum_{i=1}^{N}\sigma_{i}^{2}}$$

In the formula, *N* is the number of errors of the same reactive material and $\sigma_{i}$ is the error of different drop heights. The σ of the four reactive materials was calculated and recorded in Table 7. By comparing the σ of the four specimens, it was found that the largest σ was 89.98, which shows that the fitting function is reliable.

The ignition height of the sample is the drop height corresponding to the intersection of the fitting curve and the X-axis. Moreover, the range of the ignition height was calculated based on the average standard deviation of the flame duration of the four samples. The ignition height and range of the four specimens are recorded in Table 8.

The results show that the order of ignition height from low to high of four kinds of samples is #2 < #3 < #1 < #4. The ignition height of #2 is lower, which shows that #2 is easier to ignite. The ignition height of #4 is significantly higher than the ignition height of #3, which means that when the ratio of PTFE/Si is high, the ignition height of the reactive material will increase significantly. The reason is that the release capacity of PTFE/Al is improved by adding PTFE/Si [16], but the PTFE/Si reactive material is insensitive compared with PTFE/Al, and the addition of a higher ratio of PTFE/Si will absorb a large amount of energy released by PTFE/Al and inhibit the propagation of chemical reactions inside the reactive material, leading to an increase in the ignition height of specimens.

A drop weight test was performed on sample #3 to verify the rationality of the extrapolation method. According to the extrapolated ignition height of #3, the drop height in the experiment was selected as 50 cm. The experiment was repeated six times in total, and the ignition state in the six experiments was recorded in Table 9.

In the experiment, four samples were ignited, and two samples were not ignited. The sample could not be ignited stably at a drop height of 50 cm, which indicated that the ignition height of #3 was approximately 50 cm. The experimental result was consistent with the extrapolation result, which shows that the extrapolation method to obtain the ignition height of the reactive material is reliable.

### 3.2. Calculation of Temperature of Reaction Flame

Non-contact methods are usually used to measure flame temperature, including the total radiation method, brightness temperature method, two-color method, thermal imaging method, and multi-wavelength method. The two-color method is traditionally used to measure flame temperature [20]. The literature [21,22,23] used the two-color method to measure the flame temperature.

In this study, the two-color method was chosen to calculate the reaction flame temperature of four different specimens. Considering that the emissivity of flame radiation changes with the wavelength, two wavelengths were selected with a smaller interval in the calculation. The emissivity of flame radiation at the two wavelengths was approximately equal. Thus, the influence of the emissivity of flame radiation was eliminated. The wavelength interval was 10 nm in this study.

According to the Planck radiation law [23], the radiation intensity of an object is:

(6) $$I(\lambda ,T)=\varepsilon (\lambda )\frac{2\pi hc^{2}}{\lambda^{5}(e^{hc/\lambda kT}-1)}$$

In the formula, *h* is the Planck constant, *k* is the Boltzmann constant, and *c* is the light speed. Since the wavelength of the radiation object ranges from 200–900 nm, and the temperature ranges from 1000–3000 K, Equation (7) can be obtained:

(7) $$e^{hc/\lambda kT}\gg 1$$

Therefore, Planck’s radiation law can be rewritten as Wien’s law [24]:

(8) $$I(\lambda ,T)=\varepsilon (\lambda )\frac{2\pi hc^{2}}{\lambda^{5}e^{hc/\lambda kT}}$$

The radiation intensity *I* (λ*, T*) and *I* (λ+Δλ, *T*) of two different wavelengths emitted from one direction were obtained by the spectrometer. Then, the two radiation intensities were divided to obtain Equation (9):

(9) $$\frac{I(\lambda ,T)}{I(\lambda +\Delta \lambda ,T)}=(\frac{\varepsilon_{\lambda }}{\varepsilon_{\lambda +\Delta \lambda }}){\left(\frac{\lambda +\Delta \lambda }{\lambda }\right)}^{5}\exp(\left[-\frac{C_{2}}{T}(\frac{1}{\lambda }-\frac{1}{\lambda +\Delta \lambda })\right])$$

(10) $$C_{2}=\frac{hc}{k}$$

In the formula, $\varepsilon_{\lambda }$ and $\varepsilon_{\lambda +\Delta \lambda }$ are the emissivity of the radiation object at wavelength λ and λ+Δλ, respectively. When Δλ is very small, $\varepsilon_{\lambda }\approx \varepsilon_{\lambda +\Delta \lambda }$. The temperature of the radiation object can be calculated by the Equation (11):

(11) $$T=-C_{2}(\frac{1}{\lambda }-\frac{1}{\lambda +\Delta \lambda })/\ln\frac{\lambda^{5}I(\lambda ,T)}{{\left(\lambda +\Delta \lambda \right)}^{5}I(\lambda +\Delta \lambda ,T)}$$

**Figure 6:**
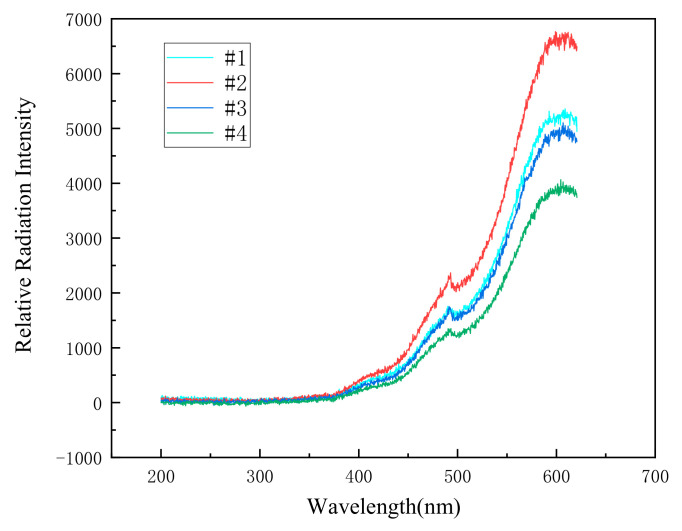
The spectrums of the reaction flame of four different specimens at a drop height of 200 cm.

According to the above formula, the flame temperature can be calculated. As shown in Figure 6, flame spectrums of four kinds of specimens were recorded by the spectrum system at a drop height of 200 cm.

The flame temperature at each set of wavelengths was calculated, then the average flame temperature could be calculated by the formula (12):

(12) $$T_{a}=\frac{1}{n}\sum_{i=1}^{n}T_{i}$$

In the formula, *n* is the number of wavelength groups and $T_{i}$ is the temperature calculated for a group of wavelengths. $T_{a}$ is considered to be the temperature measured by the spectroscopy system.

**Figure 7:**
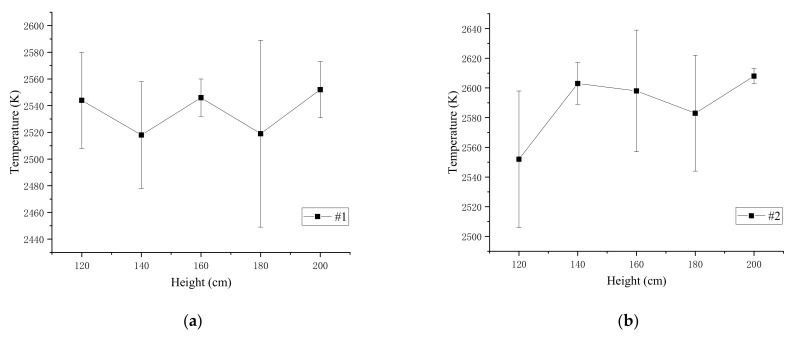

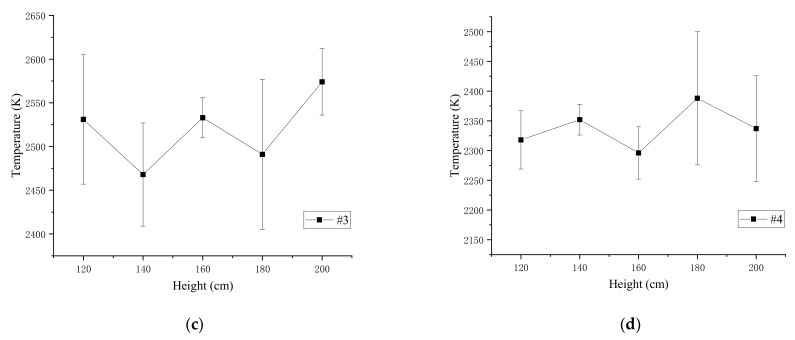
The average flame temperature and standard deviation of the four samples: (**a**) average temperature and standard deviation of #1; (**b**) average temperature and standard deviation of #2; (**c**) average temperature and standard deviation of #3; (**d**) average temperature and standard deviation of #4.

The spectrums were used to calculate the flame temperature of the four kinds of specimens at the drop height of the range of 120–200 cm, and the average temperature and standard deviation of flame calculated from three experiments are shown in Table 10. Figure 7 shows the curve of the four different specimens’ flame temperature with the drop height.

**Figure 8:**
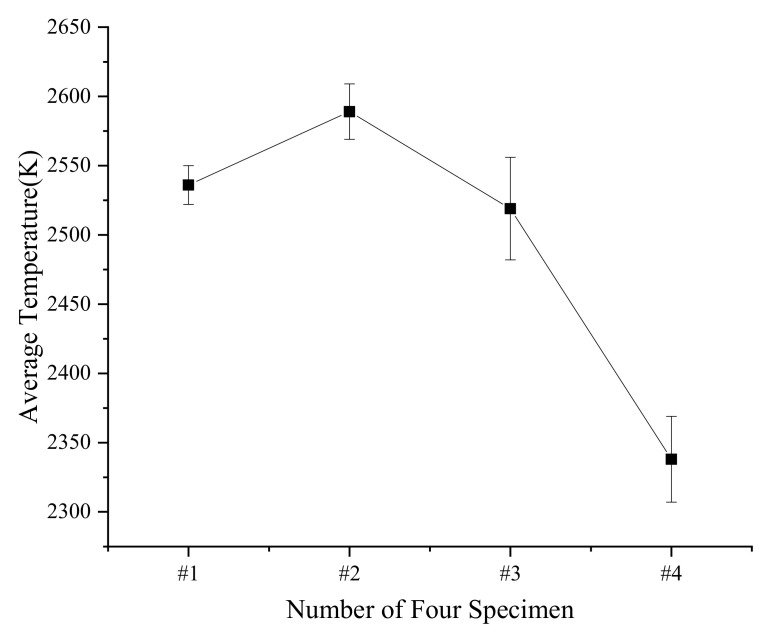
Average temperature and standard deviation of four types of specimens at different drop heights.

The results show that when the drop height is between 120 and 200 cm, the same reactive material flame temperature slightly fluctuates up and down. The standard deviations and average temperature of the four types of specimens at different heights were calculated to study flame temperature characteristics, as depicted in Figure 8 and Table 11.

The standard deviation of the #3 material is the largest, which is 37. It is considered that the flame temperature of PTFE/Al/Si/CuO does not change with the drop height. The reason is that the measured signal comes from the center of the flame. Therefore, the calculated temperature could be approximately regarded as the maximum temperature of the specimens’ reaction, which mainly depends on the reactive material composition involved in the reaction. The results show that as the mass ratio of PTFE/Si increases, the flame temperature of PTFE/Al/Si/CuO increases first and then decreases. When the mass ratio of PTFE/Si is 20%, the flame temperature is the highest, 2589 K, and when the mass ratio of PTFE/Si is greater than 30%, the flame temperature decreases rapidly.

## 4. Conclusions

In this study, PTFE/Al/Si/CuO reactive materials with four different mass ratios of PTFE/Si were prepared. The ignition height and flame temperature of four specimens were studied with drop hammer tests. The main conclusions are as follows:

1. The ignition height of four mass ratios of PTFE/Al/Si/CuO was calculated by fitting the relationship between flame duration and drop height. The results show that the ignition height first decreases and then increases with the PTFE/Si ratio increase. When the ratio of PTFE/Si is 20%, the ignition height of PTFE/Al/Si/CuO is 48.27 cm, which is the lowest among the four samples.
2. In the drop hammer tests, the flame temperature was calculated with the signal recorded by the spectrometer system. The results show that the flame temperature of the same reactive material changes little at a range height of 120–200 cm. It could be considered that the reaction flame temperature of PTFE/Al/Si/CuO does not change with the drop height within this height range.
3. With the increase of the mass ratio of PTFE/Si, the flame temperature of PTFE/Al/Si/CuO increases first and then decreases. When the mass ratio of PTFE/Si is 20%, the specimen’s flame temperature is the highest, at 2589 K.

By comparing the ignition height and the flame temperature of PTFE/Al/Si/CuO with different ratios of PTFE/Si, it was found that PTFE/Al/Si/CuO with 20% PTFE/Si has a lower ignition height and higher flame temperature, which indicates that the reactive material with this ratio has stronger damage ability.

## Figures and Tables

**Table 1:** Relative molecular mass, density, particle diameter, and manufacture of the materials.

Materials	Relative Molecular Mass	Density (g/cm^3^)	Particle Diameter (µm)	Manufacturer	Production Area
PTFE	100.0	2.1	35	DuPont	Wilmington, DE, USA
Al	27.0	2.7	5	Tianjiu metal materials Co., Ltd.	Changsha, China
Si	28.1	2.33	5	Guangzhou Nano Chemical Technology Co., Ltd.	Guangzhou, China
CuO	79.6	6.4	10	Naiou Nano Technology Co., Ltd.	Shanghai, China

**Table 2:** Number and composition ratios of four specimens.

Number	Material Mass Ratio	Remarks
#1	(PTFE/Al):(PTFE/Si):(Al/CuO) = 8:1:1	PTFE:Al:Si:CuO = 66.6:23.0:2.2:8.2
#2	(PTFE/Al):(PTFE/Si):(Al/CuO) = 7:2:1	PTFE:Al:Si:CuO = 67.0:20.4:4.4:8.2
#3	(PTFE/Al):(PTFE/Si):(Al/CuO) = 6:3:1	PTFE:Al:Si:CuO = 67.5:17.7:6.6:8.2
#4	(PTFE/Al):(PTFE/Si):(Al/CuO) = 5:4:1	PTFE:Al:Si:CuO = 68.0:15.1:8.7:8.2

**Table 3:** Mass and size of four specimens.

Number	Mass (g)	Diameter (mm)	Thickness (mm)
#1	0.21	6.04	3.07 ± 0.08
#2	0.21		3.13 ± 0.05
#3	0.21		3.14 ± 0.05
#4	0.21		3.10 ± 0.06

**Table 4:** Information about the experimental instruments.

Instrument	Model	Manufacturer	Production Area
High-speed camera	V710	Vision Research Inc	Wayne, NJ, USA
Spectrometer	Ocean Fx	Ocean Insight	Shanghai, China

**Table 5:** The average and standard deviation of flame duration at different drop height.

Height (cm)	#1 (µs)	#2 (µs)	#3 (µs)	#4 (µs)
240	2067 ± 47	2300 ± 82	2200 ± 100	1850 ± 50
200	1950 ± 50	2100 ± 100	2067 ± 205	1700 ± 100
180	1800 ± 82	1900 ± 0	1867 ± 125	1500 ± 82
160	1700 ± 100	1767 ± 125	1733 ± 47	1433 ± 125
140	1633 ± 47	1667 ± 125	1650 ± 100	1367 ± 94
120	1500 ± 82	1550 ± 100	1533 ± 47	1300 ± 82
100	1350 ± 50	1400 ± 0	1367 ± 94	1167 ± 189
80	1133 ± 249	1350 ± 100	1200 ± 100	300 ± 216
70	900 ± 141	1150 ± 100	1067 ± 47	0
60	467 ± 335	850 ± 171	683 ± 90	0
50	0	250 ± 257	167 ± 189	0

**Table 6:** The errors between the fitting value and the experimental value at different drop heights.

Height (cm)	#1 (µs)	#2 (µs)	#3 (µs)	#4 (µs)
240	−60	−164	−89	−108
200	−60	−83	−87	−51
180	19	46	1	90
160	36	95	42	86
140	2	96	50	60
120	8	90	32	−1
100	−15	79	20	−80
80	−69	−106	−72	4
70	−64	−81	−132	-
60	−74	−47	−42	-
50	-	−24	−168	-

‘-’ indicates that the reactive material was not ignited at this drop height.

**Table 7:** σ of the four reactive materials.

	#1	#2	#3	#4
σ	48.46	89.98	81.90	70.51

**Table 8:** The ignition heights and errors of the four kinds of specimens.

Number	Ignition Height (cm)	Range of Ignition Height (cm)
#1	56.29	55.64~57.11
#2	48.27	47.68~48.98
#3	50.01	49.20~50.97
#4	78.78	78.53~79.12

**Table 9:** The ignition state of the sample in the experiment.

	1	2	3	4	5	6
Ignition State	Y	Y	N	Y	N	Y

‘Y’ means that the sample was ignited, and ‘N’ means that the sample was not ignited.

**Table 10:** The average temperature and standard deviation of the flame of the four samples at different drop heights.

Height (cm)	#1 (K)	#2 (K)	#3 (K)	#4 (K)
200	2552 ± 21	2608 ± 5	2574 ± 38	2337 ± 89
180	2519 ± 70	2583 ± 39	2491 ± 86	2388 ± 112
160	2546 ± 14	2598 ± 41	2533 ± 23	2296 ± 44
140	2518 ± 40	2603 ± 14	2468 ± 59	2352 ± 26
120	2544 ± 36	2552 ± 46	2531 ± 74	2318 ± 49

**Table 11:** The average and standard deviation of reaction flame temperature of the four kinds of specimens.

	#1	#2	#3	#4
Average Temperature (K)	2536	2589	2519	2338
Standard Deviation	14	20	37	31

## Data Availability

The data can be requested from the corresponding authors.
